# Outcomes of childhood severe malaria: a comparative of study pre-COVID-19 and COVID-19 periods

**DOI:** 10.1186/s12887-023-03985-4

**Published:** 2023-04-15

**Authors:** Olayinka Rasheed Ibrahim, Michael Abel Alao, Bello Mohammed Suleiman, Olugbenga Ayodeji Mokuolu

**Affiliations:** 1 Department of Pediatrics, Federal Teaching Hospital, Katsina, Katsina State Nigeria; 2 Department of Pediatrics, University College Hospital, & University of Ibadan, Ibadan, Oyo state Nigeria; 3 Department of Pediatrics, University of Ilorin Teaching Hospital, & University of Ilorin, Ilorin, Kwara State Nigeria; 4 Department of Pediatrics and Child Health, University of Ilorin, Ilorin, Kwara State Nigeria

**Keywords:** Severe malaria, Prevalence, Children, Nigeria, COVID-19, Outcomes

## Abstract

**Background:**

The collateral damages from measures adopted to mitigate the coronavirus disease 2019 (COVID-19) pandemic have been projected to negatively impact malaria in sub-Saharan Africa. Herein, we compare the prevalence and outcomes of childhood severe malaria during the pre-COVID-19 and COVID-19 periods at a tertiary health facility in Nigeria.

**Methods:**

This was a retrospective review of cases of severe malaria admitted from 1st January to 31st December 2019 (pre-COVID-19 period) and 1st January to 31st December 2020 (COVID-19 period). We extracted relevant information, including demographics, the duration of symptoms before presentation, forms of severe malaria, and outcomes of hospitalization (discharged or death).

**Results:**

In the pre-COVID-19 period, there were a total of 2312 admissions to the EPU and 1685 in the COVID-19 period, representing a decline of 27%. In contrast, there were 263 and 292 severe malaria admissions in the pre-COVID-19 and COVID-19 periods, respectively, representing an 11% increase in the absolute number of cases. The prevalence rates were 11.4% in the pre-COVID-19 period and 17.3% in the COVID-19 period, representing an increase of 52% in the percentage differences. The mortality rate in the COVID-19 period was higher than the pre-COVID-19 period ([10.3%; 30/292 vs. 2.3%; 6/263], p 0.001). The death rate increased by 350% during the COVID-19 period. The odds ratio (OR) of a child dying from severe malaria in the COVID-19 era was 4.9 [95% confidence interval (CI): 2.008 to 11.982]. In the COVID-19 era, presentation at a health facility was also delayed (p = 0.029), as were the odds of multiple features of severe malaria manifestations (OR-1.9, 95% CI, 1.107 to 3.269; p = 0.020).

**Conclusion:**

This study shows that the prevalence of severe childhood malaria increased by as much as 11.0%, with a disproportionate increase in mortality compared to the pre-pandemic level. Most children with severe malaria presented late with multiple features of severe malaria, probably contributing to the poor hospitalization outcomes (death) observed in this study.

## Background

Malaria remains one of the most important infectious diseases in humans, with about half of the world’s population at risk [1]. Malaria’s burden has been significantly reduced thanks to various global efforts, with a 37% decrease in incidence and a 60% decrease in death between 2000 and 2015 [2]. The progress spans all the world health organization (WHO) regions, and recent data showed that Nigeria made significant progress in sub-Saharan Africa [3]. Between 2010 and 2018, malaria prevalence among children under the age of five in Nigeria fell from 42–23% [4]. This significant progress has been attributed to sustaining measures such as periodic distribution of long-lasting insecticidal nets (LLIN), improved case management, access to diagnostic services with an expanded distribution of rapid diagnostic tests reaching up to the community level, and indoor residual spraying [4, 5].

Despite Nigeria’s progress regarding the burden of malaria, the country still ranks highest in the world and contributed 27% and 32% of global cases and deaths, respectively, in 2020 [6]. In West Africa, the country accounted for half of the malaria cases in 2020, with most deaths among children. Niger, like Nigeria, is one of the 11 countries with a high burden to high impact, making it a critical strategy to combat global malaria. However, malaria cases in Niger are far fewer than in Nigeria and accounted for 3.3% of global malaria cases and 2.8% of global deaths in 2020, with a greater burden of 62% among under-five children [6].

Though there has been a reduction in global malaria infection, the herald of coronavirus 2019 (COVID-19) in late December and subsequent declaration as a pandemic in March 2020 became “a disruptor” that threatened the previous gains [7]. With a total of 660,131,952 confirmed cases and 6,690,473 deaths as of January 10th, 2023, COVID-19, caused by the severe acute respiratory syndrome coronavirus type 2 (SARS-CoV-2), remains the worst pandemic in the history of humanity [8].

Nigeria also had its share of the burden of the COVID-19 pandemic and ranked high among the countries in Africa, with total confirmed cases of 89,163 and 1302 (1.5%) mortality as of December 31st, 2020 [9]. In contrast, the Niger Republic, which shares a border with Nigeria, had far fewer cases of COVID-19, with less than 3500 confirmed cases and 110 deaths as of December 31st, 2020 [10]. Both countries experienced a series of COVID-19 waves, with the WHO dashboard as of January 3rd, 2023, indicating cumulative confirmed cases of 266,450 and 9,504 confirmed cases in Nigeria and the Niger Republic, respectively [8, 9]. Also, cumulative death as of January 3rd, 2023, in Nigeria and Niger republic were 3,155 and 315, respectively [9, 10].

COVID-19 has impacted all aspects of human life, including health care services and deliveries [8]. Consequently, the WHO and its partners projected an increase in malaria cases from 227 million in 2019 to 240 million in 2020 if the key interventions were interrupted (halting the distribution of LLIN and reducing the availability of anti-malarial drugs in the endemic regions) [11, 12]. Malaria deaths in Nigeria are expected to double, with an estimated increase of 81,000 cases [13]. Though this projection is far higher than most countries, it is not unexpected because the country has the largest burden of malaria globally [1]. The projection on the impact of COVID-19 is attributed to the indirect effect of COVID-19 mitigating measures, which include adoption of “lockdown” with restriction of movement, temporary closure of some health facilities due to absence of personal protective equipment (PPE), patients’ avoidance of health care facilities due to fear of getting infected with COVID-19, re-allocation of limited resources to combat COVID-19 with the suspension of key malaria interventions such as LLIN distribution and seasonal malaria chemoprophylaxis (SMC) [14]. Other factors that affected malaria during COVID-19 include reduced access to anti-malarials due to the closure of borders (most anti-malarials are imported from high-income countries) and the use of anti-malaria medications for COVID-19 [14, 15]. The indirect impacts of COVID-19 on malaria are well documented; there is also a possible co-infection of malaria with COVID-19, which may worsen the outcomes of either disease due to the increased release of pro-inflammatory cytokines [16]. On the contrary, the lesser burden of COVID-19 in malaria-endemic regions has also been attributed to the possible protective effects of immunomodulators from previous malaria infections [17].

Though there was a projection of increased malaria cases and subsequent confirmation in the 2021 global malaria report, which showed that malaria in endemic regions increased from 227 million in 2019 to 240 million in 2020, available literature indicated varying impacts across countries in Africa [6, 12]. Some malaria-endemic countries observed increased malaria cases, while some African countries reported decreased cases, and a few observed no changes in the burden of malaria. For instance, a retrospective study in northern Ghana that compared the pre-COVID-19 and COVID-19 periods showed a decline in malaria cases at health facilities [18]. A similar study in the malaria-endemic part of Rwanda found no change in the overall rate of uncomplicated malaria but a reduction in the proportion of severe malaria cases at the health facilities [19]. In Zimbabwe, when compared with the pre-COVID-19 period, COVID-19 was associated with a surge in malaria cases and deaths [20]. In Nigeria, a secondary analysis of data from the COVID-19 Health Services Disruption Survey in 2020 observed a reduction in the number of visits to hospitals for diagnosis of malaria as well as testing for malaria [20].

With the observed varying impact of COVID-19 malaria across Africa, the extent of the impact of COVID-19 might not have uniform applicability across sub-national levels. It is therefore important that sub-national experiences are also documented to assist in the preparation for business continuity in complex emergencies. This will be consistent with current approaches to the sub-national tailoring of interventions. Thus, we aimed to compare the prevalence and outcomes (hospitalizations, deaths, and discharge) of childhood severe malaria admitted between the pre-COVID-19 period (2019) and the COVID-19 period (2020) at a tertiary health facility in northern Nigeria. Our secondary outcome was also to compare the profile and index of severity between the two timelines.

## Methods

### Study design and settings

This was a retrospective review of severe malaria cases admitted from 1st January to 31st December 2019, otherwise referred to as the pre-COVID-19 period and January 1st to 31st December 2020 (COVID-19 period) at Federal Teaching Hospital, Katsina, Nigeria. The hospital is a 700-bed tertiary health facility located in north-western Nigeria and receives referrals within the state, parts of adjoining states, and the Niger republic. This study took place in the emergency and pediatric wards of the hospital. The hospital has a dedicated COVID-19 isolation and treatment center, which is separate from other hospital structures including the pediatric wards. In addition, during the peak of the first wave of COVID-19 in Nigeria, the hospital remained open including the pediatric units and ensured minimal disruption of services.

### Study population

This study involved children aged 14 years and below with the diagnosis of severe malaria during the study period (1st of January 2019 to 31st December 2020).

### Case definition for severe malaria

The definition of severe malaria was based on the 2015 WHO guidelines for severe malaria [21]. In brief, a case of severe malaria was defined as evidence of parasitological confirmation and the presence of life-threatening features, which include any of the following: “Impaired consciousness” was defined as a Glasgow score of less than 11 or a Blantyre coma score of less than 3 for younger children; “prostration” was defined as generalized weakness (a child was unable to sit, stand, or walk without assistance); “multiple convulsions” was defined as a child having more than two episodes of convulsion within 24 h; “Acidosis” was defined as a plasma bicarbonate level of less than 15 mmol/L; “hypoglycaemia” referred to blood or plasma glucose of less than 2.2 mmol while “severe anemia” was defined as hemoglobin concentration less than or equal 5 g/dl or packed cell volume of less than or equal to 15% in children < 12 years of age and less than 7 g/dl or packed cell volume of 20% in those aged 12 years; “acute kidney injury” was defined as plasma or serum creatinine 3 mg/dl or blood urea > 20 mmol/l while “jaundice” was defined as the plasma or serum bilirubin > 50 umol/l or 3 mg/dl; “pulmonary oedema” was defined as radiologically confirmed or oxygen saturation < 92% on room air with a respiratory rate greater than 30/minute, or age appropriate tachypnoea often with chest indrawing and crepitations on auscultation; “significant bleeding” referred to recurrent or prolonged bleeding from the nose, gums or venepuncture sites; hematemesis or melena; “Compensated shock” was defined as capillary refill greater or equal three seconds but no hypotension, and “decompensated shock” was defined as systolic blood pressure < 70 mm Hg with evidence of impaired perfusion (cool peripheries or prolonged capillary refill). “Hyperparasitemia” was defined as plasmodium falciparum parasitemia > 10%.

The parasitological confirmation in this study was based on the child being tested positive with “rapid diagnostic test” (RDT) kits and/or the presence of malaria parasites on light microscopy.

### Period of study

The pre-COVID-19 period lasted from January 1, 2019 to December 31, 2019.The COVID-19 period lasted from January 1, 2020, to December 31, 2020, as the first case of COVID-19 was confirmed in Nigeria on February 27, 2020 [22].

### Sample size

Using the previous prevalence of 27.9% reported in the study center and a prevalence of 49.5% in the region from the Nigeria Malaria Indicator Survey, we calculated a minimum size of 198 children for each year using an online sample size calculator (https://epitools.ausvet.com.au/twoproportions) at a power of 95% and 99% confidence level [23, 24].

### Inclusion criteria

Children with parasitological confirmed severe malaria were included in the study.

### Exclusion criteria

We excluded the following children from the study: children younger than three months; the presence of comorbidities such as sickle cell disease and chronic kidney disease; and those with underlying severe acute malnutrition.

### Outcomes measured

The primary outcomes of this study were to determine and compare the prevalence and hospitalization outcomes (length of stay from the day of admission, deaths, or discharge) between the pre-COVID-19 and COVID-19 periods among children with severe malaria. The secondary outcomes were to describe and compare the profile and index of severity of severe malaria between the two periods.

### Data extraction

The data of children with severe malaria admitted from January 1, 2019 to December 31, 2020 was extracted from the electronic health records. Variables extracted included age, sex, duration of symptoms before presentation at our health facility, duration of hospitalization, forms of severe malaria, date of admission, date of outcomes of hospitalization, and outcomes of hospitalization (defined as death or discharge).

### Data analysis

The data were entered into an Excel spreadsheet and exported into SPSS version 25 for analysis. The age, duration of symptoms, and length of hospitalization were not normally distributed and were summarized as medians with an interquartile range. In addition, the three variables were compared using Mann-Whitney U between the pre-COVID-19 and COVID-19 periods. The discrete variables, such as features of severe malaria, sex, and hospitalization outcomes, were summarized using frequency tables and compared using Chi-square. In addition, we compared the cases and hospitalization outcomes between the two periods using the COVID-19 period as the exposure events with the odds ratio and a 95% confidence interval. The p-value was set at 0.05 for all the statistical tests.

## Results

### General characteristics of the study population

In the pre-COVID-19 period, there were a total of 2312 admissions to the emergency and pediatric wards, and there were 1685 in the COVID-19 period, representing a decline of 27%. The median (interquartile range) age of the study children was 4.0 (2 to 7.3) years, with a range of 0.40 to 14 years. The age of children admitted with severe malaria was younger in the COVID-19 period compared with the pre-COVID-19 period, p = 0.002 (Table 1). Out of the 555 children with severe malaria, 308 were male (55.5%). There were no significant differences in the genders between the pre-COVID-19 and COVID-19 periods (Table 1).

**Table 1:** Demographic characteristics of the study children

Variables	Total n = 555	pre-COVID-19 period n = 263	COVID-19 period n = 292	${\text{U/}}{\chi ^2}$	p
**Age (years)**					
**Median (IQR)**	4 (2.1 to 7.3)	5 (3.0 to 8.0)	4 (2.0 to 7.0)	32661.50^U^	0.002
**Less than 5**	293 (52.8)	125 (47.5)	168 (57.5)	7.235	0.027
**5 to 10**	202 (36.4)	102 (38.8)	100 (34.2)		
**Greater than 10**	60 (10.8)	36 (23.7)	24 (8.3)		
**Sex**					
**Male**	308 (55.5)	139 (52.9)	169 (57.9)	1.415	0.266
**Female**	247 (44.5)	124 (47.1)	123 (42.1)		

IQR: Interquartile range; U: Mann-Whitney U test. Pre-COVID-19 (2019); COVID-19 periods (2020)

### Prevalence of malaria

**Fig. 1:**
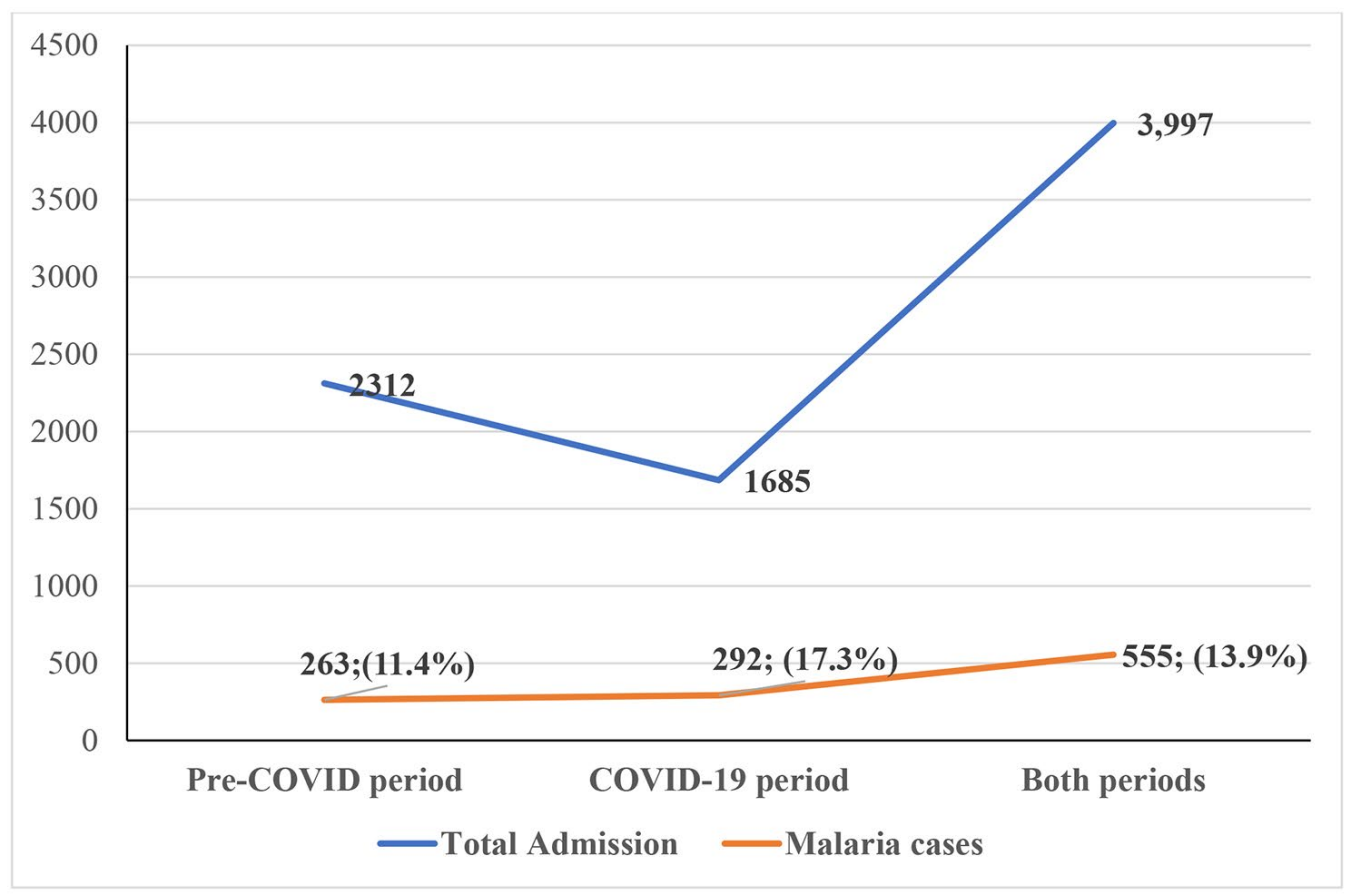
Prevalence of severe malaria in the pre-COVID-19 period (2019) and COVID-19 period (2020)

**Fig. 2:**
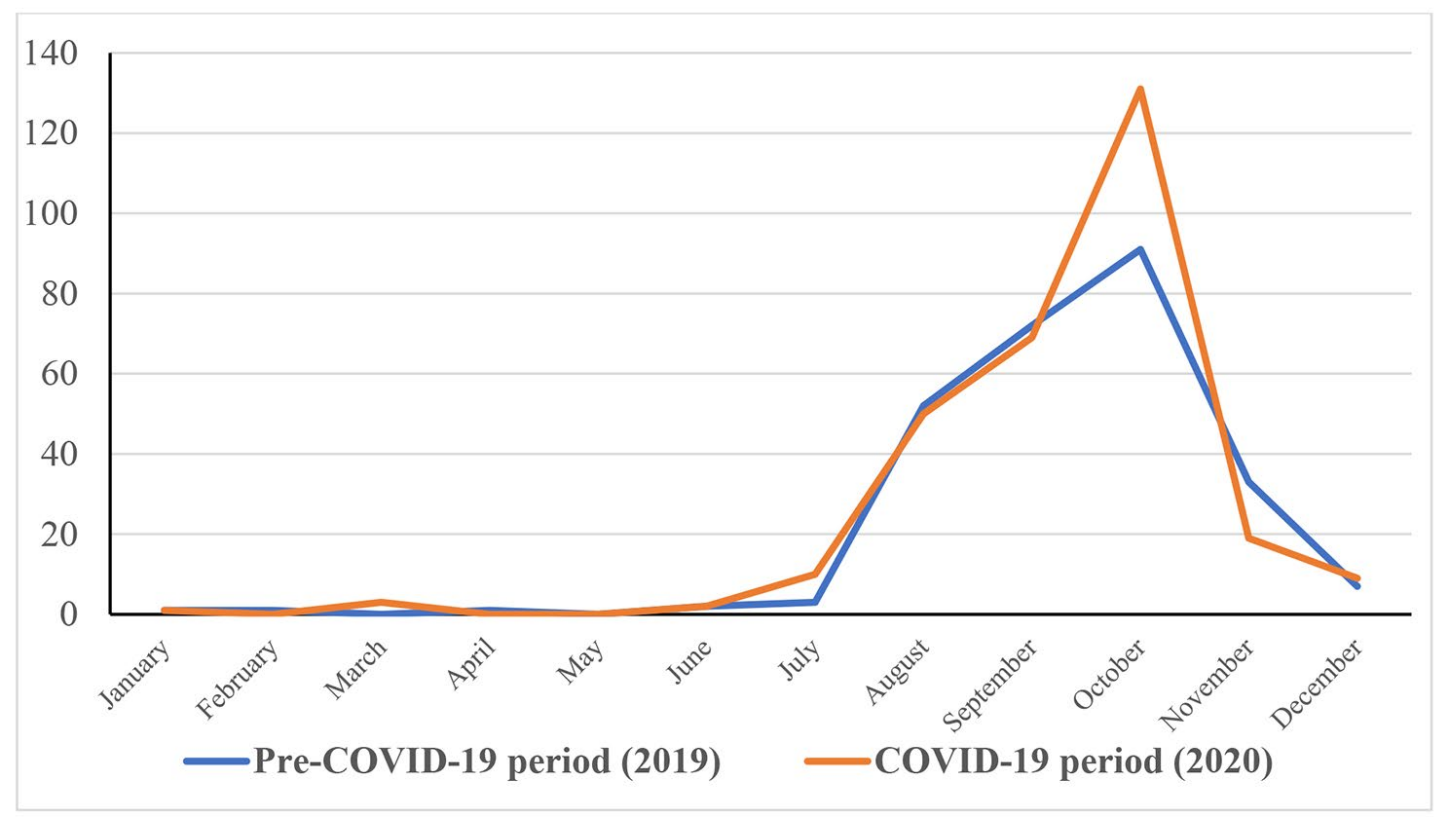
Trends of severe malaria in the pre-COVID-19 (2019) and COVID-19 (2020) periods

The overall prevalence of severe malaria over the two-year study period was 13.9% (555/3,997). There were 263 and 292 severe malaria admissions in the pre-COVID-19 and COVID-19 periods, respectively, representing an 11% increase in the absolute number of cases. The prevalence rates were 11.4% (263/2312) before COVID-19 and 17.3% (292/1685) after COVID-19, representing a 52.0% increase in percentage differences (Fig. 1). The odds ratio of malaria during COVID-19 compared with the pre-COVID-19 period was 1.633 (95% confidence interval, 1.364 to 1.955). The trends of prevalence in the two periods follow a similar pattern, but with a higher surge in the month of October in the COVID-19 period compared with the pre-COVID-19 period (Fig. 2).

### Hospitalization outcomes

The overall case fatality rate over the two periods was 6.5% (36/555). The mortality rate in the COVID-19 period was significantly higher than in the pre-COVID-19 period ([10.3%; 30/292 vs. 2.3%; 6/263], p 0.001) (Table 2). The percentage increase in deaths during the COVID-19 period when compared with the pre-COVID-19 period was 350.0%. The odds of child death from severe malaria in the COVID-19 period compared with the pre-COVID-19 period were 4.9, 95% CI 2.008, 11.982 (Table 3). The highest death rate was in the under-five age group (7.2%), though it was not statistically different from the other age groups (Tables 2 and 3). There was a presentation delay amongst children admitted during the COVID-19 period compared with the pre-COVID-19 period, p = 0.029 (Table 4). However, the duration of hospitalization (defined from the point of admission until discharge or death) was shorter during the COVID-19 period compared with the pre-COVID-19 period (Table 4). Compared with the pre-COVID-19 period, the proportion of children admitted with two or more features of severe illness was significantly higher during the COVID-19 period (Table 5). The most common features of severe malaria in the study were cerebral malaria (27.4%), multiple convulsions (15.9%), and severe anemia (13.2%). Whereas the pattern of features of severe malaria between the two periods of the study was comparable, more children were admitted with severe anemia in the COVID-19 period (Table 5).

**Table 2:** Hospitalization outcomes of children admitted with severe malaria during the COVID-19 period compared with pre-COVID-19 period

Variables	No of cases	No of deaths	Case fatality rate (%)
**Period of study**			
Both*	555	36	6.5
pre-COVID-19	263	6	2.3
COVID-19	292	30	10.3
**Age group** Both*			
Less than 5 years	293	21	7.2
5–10 years	202	11	5.4
Greater than 10 years	60	4	6.7
**Age group-** **Pre-COVID-19**			
Less than 5 years	125	4	3.2
5–10 years	102	2	2.0
Greater than 10 years	36	0	0
**Age group-** **COVID-19**			
Less than 5 years	168	17	10.1
5–10 years	100	9	9.0
Greater than 10 years	24	4	16.7

Pre-COVID-19 period (2019), COVID-19 period (2020). *Both (pre-COVID-19 and COVID-periods)

**Table 3:** Bivariate analysis of factors that are associated with death

Variables	OR ratio	p value		95% CI	
				Lower	Upper
**Periods**					
pre-COVID-19	1				
COVID-19	4.905	< 0.001		2.008	11.982
Age group (years)*					
Greater than 10	1				
5–10	0.806	0.721		0.247	2.632
Less than 5	1.081	0.890		0.357	3.271
**pre-COVID-19**					
Age group (years)					
Greater than 10	1				
5–10	1.816	0.702		0.085	38.727
Less than 5	2.704	0.662		0.142	51.406
**COVID-19**					
Age group (years)					
Greater than 10	1				
5–10	0.495	0.278		0.138	1.767
Less than 5	0.563	0.342		0.172	1.841

*Both study periods; OR-odds ratio; CI-confidence interval

**Table 4:** Comparison of duration before and during hospitalization for the study periods

Variables	Total	pre-COVID-19 period	COVID-19 period	U		p value
**Duration before presentation**	3 (2 to 4)	3 (2 to 4)	3 (2 to 5)	26712.50^U^		0.029
**LOH**	3 (1 to 6)	4 (2 to 12)	2 (1 to 3)	26786.50^U^		< 0.001
**Index of severity**						

LOH: Length of hospitalization; pre-COVID-19 (2019) period, COVID-19 (2020) period, U-Mann-Whitney U test;

**Table 5:** Bivariate analysis index of severity and periods of the study

Variables	Total	pre-COVID-19 period	COVID-19 period	OR	p value	95% CI	
						Lower	Upper
**Features of severe malaria**							
One feature	391 (70.5)	206 (78.3)	185 (63.4)	1			
Two features	99 (17.8)	33 (12.5)	66 (22.6)	2.227	0.001	1.402	3.537
Three or more features	65 (11.7)	24 (9.1)	41 (14.0)	1.902	0.020	1.107	3.269

OR-odds ratio; CI-Confidence interval.

**Table 6:** Features of severe malaria in the study children

Features of severe malaria	Both periods		Pre-COVID-19 period		COVID-19 period	
	n = 555	%	n = 263	%	n = 292	%
**Cerebral malaria**	152	27.4	80	30.4	72	24.7
**Multiple convulsion**	88	15.9	45	17.1	43	14.7
**Severe anemia**	73	13.2	31	11.8	42	14.4
**Prostration**	64	11.5	45	17.1	19	6.5
**Hemoglobinuria**	38	6.8	15	5.7	23	7.9
**Cerebral malaria, hemoglobinuria**	30	5.4	8	3	22	7.5
**Severe anemia, hemoglobinuria**	27	4.9	7	2.7	20	6.8
**Severe anemia, cerebral malaria**	15	2.7	7	2.7	8	2.7
**Cerebral malaria, severe anemia, hemoglobinuria**	14	2.5	4	1.5	10	3.4
**Multiple convulsions, hemoglobinuria**	10	1.8	3	1.1	7	2.4
**Shock**	9	1.6	3	1.1	6	2.1
**Severe anemia, multiple Convulsions**	8	1.4	2	0.8	6	2.1
**Acute kidney injury**	7	1.3	2	0.8	5	1.7
**Cerebral malaria, acute kidney injury**	7	1.3	3	1.1	4	1.4
**Severe anemia, hemoglobinuria, acute kidney injury**	4	0.7	3	1.1	1	0.3
**Multiple convulsions, hemoglobinuria, severe anemia**	4	0.7	1	0.4	3	1
**Cerebral malaria, hemoglobinuria, hyperbilirubinemia, hypoglycaemia, severe anemia**	3	0.5	3	1.1	0	0
**Hypoglycaemia**	2	0.4	1	0.4	1	0.3

**Pre-COVID-19 (2019); COVID-19 (2020)**

## Discussion

The impact of COVID-19 on healthcare systems and the top three infectious diseases of tuberculosis, HIV, and malaria has been that of a possible reversal of progress in the global efforts to control the diseases [25]. This study showed that the cases of severe malaria increased by about 11% during COVID-19. This finding supported a recent global malaria report that found a rise in global malaria cases, with Nigeria leading the way [6]. A retrospective study in Zimbabwe also observed increased cases of malaria, though the data did not disaggregate the forms of malaria and involved the whole population [20]. In contrast, a study in Ghana that compared similar periods observed a reduction in the cases of malaria among under-five [18]. In Rwanda, a study that also compared similar periods to this current study did not observe any change in the cases of severe malaria [19]. It is worthy of note that the site of this present study falls under the malaria endemic zone in Nigeria, with high seasonal variability. The increase in severe malaria cases observed in this study may be related to the indirect impact of COVID-19 on malaria prevention and control strategies in the country, such as postponement of LLIN distribution, delayed deployment of seasonal malaria chemoprophylaxis (SMC), and possible restriction of access to health at secondary and primary healthcare facilities due to a lack of personal protective equipment (PPE) and uncertainty surrounding the disease [14]. Though COVID-19 is less common in children with good outcomes compared with adults in Nigeria, [26, 27], a possible co-infection may also contribute to the increased severity of malaria observed in this study.

Also worthy of note is the fact that the trends of severe malaria prevalence in the two periods follow a similar pattern, but with a higher surge in the month of October in the COVID-19 period. The trends in this study are similar to the pattern we earlier reported and called for sustained use of SMC adopted by the country under which the state falls [23]. While the country can be commended for putting in place a number of mitigating actions to ensure that the needed interventions were delivered, the COVID-19 and response still resulted in significant delays in the timing of the interventions. With the reported seasonality of malaria in the area, such delays implied that the population was not adequately protected from the potential impact of the malaria season. Hence, the surge in malaria cases observed in this study.

This study also found children with severe malaria had delayed presentation (duration of symptoms before presentation at the hospital) at the health facility and a shorter hospitalization during COVID-19. Though there is a paucity of local studies that examine delayed presentation and COVID-19 among ill children, a multi-national survey in high-income countries showed significant delays before presentation among children and adolescents, mainly due to the fear of COVID-19 [28]. This observation in this study affirmed one of the postulates regarding the indirect impact of COVID-19 on children attributed to the fear of contracting SARS-CoV-2 by the parents and/or their children. It is therefore possible that patients only presented to the hospital when death was imminent, and the patients can no longer be nursed at home. This delayed presentation may also have contributed to the high mortality rate observed during COVID-19 and suggests the need for a pragmatic approach to child care during a pandemic that ensures parents can assess care early without fear when their children are sick.

Based on the index of severity, more children were admitted with two or more features during COVID-19. In addition, a higher number of children presented with severe anemia during COVID-19. These findings probably reflect the delayed presentation and subsequent progression of the illness among the children from uncomplicated to severe forms of malaria. A Canadian study found that delayed presentation was associated with sicker children [29]. These findings may also reflect the multiplier effects of the various indirect impacts of COVID-19 on childhood malaria, such as reduced access to medications and reduced access to healthcare facilities due to the closure of some health facilities in the absence of PPE [13]. These findings also show that if access to diagnosis and treatment is delayed, children are more vulnerable to severe forms of malaria with more components and a poorer outcome.

Our study also showed that deaths from severe malaria increased to about 350% in the COVID-19 period, which is higher than the projection by WHO [11]. Furthermore, the risk of a child dying from severe malaria increased fivefold during COVID-19. in Zimbabwe, the death rate from malaria tripled during COVID-19 [20]. In Sierra Leone, no change was observed in the mortality rate among under-five children with severe malaria during the pre-COVID-19 and COVID-19 periods [30]. The higher death rate obtained in this study compared with projections by the WHO may be due to the fact that this is a hospital-based study and may likely have sicker children. The differences concerning the Sierra Leone study may be related to the study period and differences in the malaria country profiles of the two countries [6]. The present study spanned a complete calendar year for both COVID-19 and pre-COVID-19 periods, compared with Sierra Leone, which was limited to January through May. In addition, variation in the profile of malaria in the two countries may have also accounted for the observed differences. In addition, a few of the children may have missed co-infections, which probably contributed to the increased severity of their illness and the poorer outcomes obtained in this study. However, it is worthy of note that a few studies also observed no increased mortality in the presence of co-infection, which calls for further study [31, 32]. The import of our findings of a higher death rate in this study shows that the deaths from severe malaria during COVID-19 may be far higher than reported, hence the need for a review of the country data, especially at the tertiary health facilities that manage the most complicated forms of malaria in Nigeria.

Although this study has a large sample size (555 children with severe malaria) and spanned two calendar years, pre-COVID-19 and COVID-19, there are some limitations. COVID-19 screening was not routinely done for children with fever in our center; however, a few (10) tested negative for COVID-19 during the first wave in Nigeria. Our study period for COVID-19 also spanned from January to December 2020. The first case confirmation in Nigeria was on February 27th, 2020 [33]. The WHO dashboard, including Nigeria, has since been updated to reflect January 2020, the month of spread outside China [8]. It is also worth noting that according to our data, only one case of severe malaria was diagnosed before March 2020 from our COVID-19 data. In addition, this is tertiary health facility-based data and may not reflect the community levels of childhood malaria, which are mostly uncomplicated malaria. Also, our findings may not be generalizable to the whole country.

In conclusion, this study shows that the burden of childhood severe malaria (prevalence) increased by as much as 11.0% with a disproportionate increase in mortality compared with the pre-pandemic level. Most of the children with severe malaria presented late with multiple features of severe malaria, which probably contributed to the poor hospitalization outcomes (death) observed in this study. We recommend the need for a clear country policy and pragmatic approaches that will ensure minimal interruption of health care services and programs that address the leading cause of childhood death, such as malaria, in the face of a pandemic.

## Data Availability

The datasets generated during the current study are not publicly available due to Federal Teaching Hospital Katsina policy on data management, but are available from the corresponding author on reasonable request (Olugbenga A Mokuolu, Department of Pediatric, University of Ilorin, Ilorin, Nigeria).

## Abbreviations

COVID-19: Coronavirus disease 2019
FTH: Federal Teaching Hospital
LLIN: long lasting insecticidal net
PPE: personal protective equipment
SMC: Seasonal malaria chemoprophylaxis
